# Workplace violence toward resident doctors in public hospitals of Syria: prevalence, psychological impact, and prevention strategies: a cross-sectional study

**DOI:** 10.1186/s12960-020-00548-x

**Published:** 2021-01-07

**Authors:** Okbah Mohamad, Naseem AlKhoury, Mohammad-Nasan Abdul-Baki, Marah Alsalkini, Rafea Shaaban

**Affiliations:** 1 Faculty of Medicine, Tishreen University, Latakia, Syria; 2 University of Hama College of Human Medicine, Hama, Syria; 3 Faculty of Medicine, Albaath University, Homs, Syria; 4 Faculty of Medicine, Al-Andalus University for Medical Sciences, Tartus, Syria; 5 Faculty of Medicine, Tartus University, Tartus, Syria

**Keywords:** Workplace violence (WPV), Resident doctors, Public hospitals, Syria

## Abstract

**Introduction:**

Workplace violence (WPV) against healthcare workers is a common and daily problem in hospitals worldwide. Studies in different countries indicated that exposure to WPV potentially impacts the psychological status of healthcare workers. However, there is a paucity of studies approaching this issue in the Syrian healthcare system.

**Objectives:**

This study had three objectives: (1) to estimate the prevalence of violence against resident doctors in Syria, (2) to examine the association between WPV and resident doctors’ psychological stress, sleep quality, depression, and general health and (3) to suggest approaches to tackle this problem from the resident doctors' perspectives.

**Methods:**

A cross-sectional study was conducted in 8 out of 14 provinces, and covered 17 out of 56 accessible functioning hospitals in Syria. Data were collected using anonymous, self-administered questionnaires during February 2020. A total of 1226 resident doctors volunteered to participate in the study. Finally, 1127 valid questionnaires were used in the final data analysis. The overall response rate was 91.92%.

**Results:**

A total of 955 participants (84.74%) reported exposure to WPV in the 12 months prior to the study. In specific, 84.74% exposed to verbal violence and 19.08% to physical violence. Patients’ associates were the predominant aggressors in both verbal and physical violence (*n* = 856; 89.63%, *n* = 178; 82.79%, respectively). Most resident doctors (87.31%) suggested enacting more legislation to protect doctors as the best solution to reduce WPV. Verbal and physical violence showed a significant positive correlation with each item of depression and stress, and a significant negative correlation with both subjective sleep quality and subjective health.

**Conclusion:**

Workplace violence against resident doctors in Syria is highly common. Therefore, policymakers, hospital managers, and supervisors should work collaboratively in order to minimize WPV and ensure resident doctors’ safety and psychophysical stability.

## Introduction

Workplace violence (WPV) describes deliberate physical, psychological, sexual, and other acts against someone at work that may risk his/her health or even cause death [1]. Regarding this definition, almost all workers are susceptible to experiencing WPV as part of their job. However, WPV in health settings has reached an epidemic proportion [2], and healthcare workers face a far higher risk of being injured during their job than workers in other occupational groups [2–4].

The prevalence rate of WPV differs considerably between countries and environments of practice [5]. One survey, for instance, by the U.S. Bureau of Labor Statistics reported that healthcare workers experienced 73% of violence-related injuries and illnesses in 2018 [6]. A research in China estimated WPV prevalence towards healthcare workers at 56.4% [7]. Other studies in the Arabic region can be found too. Namely, a study in Iraq revealed a high prevalence (more than 85%) of violence toward healthcare workers [8].

In the contexts of wars and crises, violence toward healthcare workers is expected to escalate due to extreme tension and hostile conditions. This has been seen in the literature as healthcare workers were subjected to overt attacks and threats in times of war [8–10]. Furthermore, the indirect effects of war represented by the lack of supplies and human resources could contribute to violence accidents [8].

The Syrian crisis started in 2011 and has affected Syrians' life in every aspect [11]. Destruction had entailed a high proportion of health facilities, and many healthcare workers had left the country, had been injured, or even lost their lives [12]. Unfortunately, the shortage of medical professionals and the increased weight of war placed a tremendous burden on the remaining doctors, requiring them to deal with an increased number of patients and worsening the quality of care provided, thereby, doctors are becoming more prone to WPV and its consequences. Also, the lack of medical utilities and medications due to sanctions imposed on Syria and the indirect effect of war has led to deterioration of the quality of healthcare, which could lead to patients' dissatisfaction with health services [13].

The effects of WPV have been seen to contribute to declined job performance [14], higher stress levels [15], depression [16], and impaired sleep quality [17]. Studies to understand this issue are important in order to provide a safer work environment, enhance the quality of healthcare services, and for patients satisfaction.

Most of the resources about WPV against healthcare workers in Syria were news, editorials or correspondence articles with no major research studies. Therefore, this study aims to estimate the prevalence of violence against resident doctors in Syria, investigate the association between exposure to WPV and health-related outcomes in terms of psychological stress, sleep quality, depression, and the overall subjective health of Syrian resident doctors, and suggest approaches to tackle this problem from the resident doctors' perspectives.

## Methods

### Sample and procedure

A cross-sectional study was conducted within one month duration in February 2020 upon public Syrian hospitals that offer one of the training residency programs approved by the Syrian Commission of the Medical Specialties: Ministry of Higher Education (MoHE), Ministry of Health (MoH), and Ministry of Defense (MoD).

The selection was designed to represent all of the country's geographical regions (north, west, middle, east, and south). Each region was represented by two provinces with the exception of the north and east, which were represented by one province owing to war conditions. Hospital selection was mainly based on accessibility and full functionality. We selected the main MoH hospital in each province, which is characterized by the highest number of medical specialties available relative to other hospitals, except for Damascus, where we selected two hospitals since it is the capital city and has more hospitals than other cities. MoHE hospitals are only situated in four provinces (Lattakia, Aleppo, Damascus, and Rural Damascus). Lattakia contained one MoHE hospital. Two main MoHE hospitals were selected from each of Damascus and Aleppo. Accessible functioning MoD hospitals are located in three provinces (Homs, Lattakia, and Damascus); the main hospital was chosen from each province. Ultimately, the resident doctors were recruited from 17 out of 56 accessible functioning hospitals (MoH: 9/35, MoHE: 5/12, and MoD: 3/9) located in 8 provinces (Damascus: 5, Lattakia: 3, Tartous: 1, Hama: 1, Homs: 2, Aleppo: 3, Al-Hasakah: 1, and Al-Sweda: 1). Hence, each residency program was represented by approximately a third of the overall number of hospitals.

Anonymous and self-administered questionnaires were distributed and collected by researchers and contributors (resident doctors and senior medical students) who were trained to understand the study objectives and answer participants’ questions. The sampling criteria of resident doctors were as follows:

- The resident doctor did not help in the questionnaire distribution.
- The resident doctor is in his second year of residency training or more.
- The doctor has not yet finished his residency training.

The resident doctors were approached in the hospital cafeteria, following work shifts, and on an individual basis.

The contributors informed the participants that filling out the questionnaire could take up to 5–10 min, reminded them to pay attention to answer all the questions, and were available to explain any unclear questions to participants.

A total of 1226 resident doctors volunteered to participate in the study. 99 questionnaires were incomplete or poorly answered (for example, selecting multiple responses to one-response items). Finally, 1127 out of 1226 valid questionnaires were used in the final data analysis. The overall response rate was 91.92%.

### Questionnaire

All questions were administered to resident doctors in Arabic language as Arabic is the native language of the Syrian resident doctors.

#### Demographic questionnaire

The demographic questionnaire gathered data on age, gender, specialty, year of residency, residency province, residency program, financial income, and marital status.

#### Workplace violence against resident doctors

The questionnaire was built based on review of the Workplace Violence in the Health Sector Country Case Studies Research Instruments Questionnaire [18] and questionnaires that had been used in pertinent studies [19, 20].

Participants were asked to report on their experiences over the past 12 months, including the following items: (1) how often have you suffered from verbal violence in the past 12 months? (2) How often have you suffered from physical violence in the past 12 months? The response categories for each item were: never, rarely (< 12 times in the past 12 months), sometimes (1–3 times per month), often (1–5 times per week), and always (almost every day). The incidence of a specific form of violence was dichotomized as yes (ranging from rarely to always, and it was coded as ‘1′) or no (never, and it was coded as ‘0′).

In our questionnaire, verbal violence was defined as using offensive language, yelling, or screaming with the intent to offend or frighten. Physical violence was defined as a physical assault or any attempt at a physical attack. Physical assaults included behaviors, such as hitting, pulling, pushing, slapping, hair pulling, or arm twisting with intent to cause bodily harm.

We also investigated the following domains related to each type of violence (if the resident doctor had experienced that type of violence):

- Aggressor sex
- Aggressor
- Accident time
- Accident location
- Causes of the event: ‘Refusing patient’s admission to the hospital’ was defined as doctors may refuse to admit patients due to non-serious injury, overloaded sections and priorities, or unavailable services.

#### Strategies to prevent workplace violence

This section was prepared by reviewing relevant studies [19, 20] and in consultation with the senior author to make necessary changes according to our requirements. We used one question: ‘What are the necessary procedures to minimize the number of violent acts as much as possible?’ including the following answers: educational lectures to increase the awareness of workplace violence, training of resident doctors using workplace violence prevention programs to handle WPV, video recording, enacting more legislation to protect doctors, increasing security guards, increasing staff number, restricting visitors' access to hospital departments, and violence reporting system. Furthermore, participants with no experience of workplace violence were given the chance to select answers concerning the most effective measures to reduce workplace violence.

#### Subjective health and depression

We measured subjective health and depression using the Copenhagen Psychosocial Questionnaire II (COPSOQ II) [21]. Subjective health was estimated by one global item (‘In general, would you say your health is (4) excellent, (3) very good, (2) good, (1) fair, (0) poor). Depression scale had four items. The introduction asked participants to think about how often in the past 4 weeks they had experienced each item. The COPSOQ II measure of depression was designed to measure the level of depressive symptoms experienced by workers rather than to diagnose clinical depression. An example item is, ‘How often have you had a bad conscience or felt guilty?’ All items were asked on a scale of 1 (not at all), 2 (a small part of the time), 3 (part of the time), 4 (a large part of the time), or 5 (all the time).

#### Psychological stress

In this study, the 2 single-item measures were used to measure the level of stress of the participants [22]. The baseline questionnaire included 2 questions on stress. The first question measured the ability to handle stress, phrased ‘On a scale of 1 to 6, how would you rate your ability to handle stress?’ (from 1 for ‘I can shake off stress’ to 6 for ‘Stress eats away at me’). The second question ascertained perceived stress, phrased ‘In the past year, how would you rate the amount of stress in your life (at home and at work)?’ (from 1 for ‘no stress’ to 6 for ‘extreme stress’).

#### Sleep

Subjective sleep quality was measured by using one item [23] (‘During the past month, how would you rate your sleep quality overall?’). The response format ranged from very bad (0) to very good (3).

### Statistical analysis

Our data were entered in Microsoft Excel software and the statistical analysis was carried out using the Stata software package (version 6; Stata Corp, College Station, TX, USA). Data were expressed as percentages. The Chi-square test was used to analyze differences between groups. Spearman’s correlation coefficient was used for ranked variables to describe the relationship between each type of violence and psychological outcomes. We considered *p *value < 0.05 to be significant.

## Results

### Socio-demographic characteristics of the participants

Table 1 presents the socio-demographic characteristics of respondents. Most participants were male (60.78%), half of them (49.33%) aged 27–29 years, and the majority of them were from internal medicine (*n* = 365; 32.39%) followed by surgery (*n* = 238; 21.12%). Second and third-year resident doctors formed 63.26% of the participants. 521 (46.23%) of the participants were related to the Ministry of Higher Education (MoHE), 467 (41.44%) were related to the Ministry of Health (MoH), and 139 (12.33%) were related to the Ministry of Defense (MoD). The majority of participants (65.39%) were single and 40.82% were from Damascus city. For 32.92% of the participants, their income was from their job salary and family support, and for 16.95%, the salary was their only income.

**Table 1:** Socio-demographic characters of resident doctors in Syria, (*n* = 1127)

Variables	Frequency	Percentage
Age		
24–26	449	39.84
27–29	556	49.33
30–35	122	10.83
Sex		
Male	685	60.78
Female	442	39.22
Specialty		
Internal medicine	365	32.39
Surgery	238	21.12
Pediatrics	120	10.65
Obstetrics	101	8.96
Dermatology	81	7.19
Ophthalmology	63	5.59
ENT	59	5.24
Emergency medicine	25	2.22
Anesthesiology	22	1.95
Radiology	20	1.77
Oncology	16	1.42
Pathology	11	0.98
Family medicine	4	0.35
Psychiatry	2	0.18
Year of residency		
Second	360	31.94
Third	353	31.32
Fourth	239	21.21
Fifth	135	11.98
Sixth	32	2.84
Seventh	8	0.71
Related authority		
Ministry of Higher Education	521	46.23
Ministry of Health	467	41.44
Ministry of Defence	139	12.33
Residency province		
Damascus	460	40.82
Latakia	236	20.94
Aleppo	188	16.68
Homs	108	9.58
Tartous	43	3.82
Hama	36	3.19
Al-Hasakah	33	2.93
Al-Sweda	23	2.04
Marital status		
Married	390	34.61
Not married	737	65.39
Financial income (multiple answers)		
Salary	985	87.4
Family support	605	53.68
Extra work	456	40.46

### Percentages of exposure to verbal and physical violence by demographic characteristics

The percentages of exposure to verbal and physical violence by socio-demographic characteristics are shown in Table 2. A total of 955 participants (84.74%) reported exposure to at least one type of WPV in the 12 months prior to the study. In specific, 84.74% of the subjects reported exposure to verbal violence and 19.08% to physical violence. This shows that all physical violence incidents were preceded by verbal violence. With regard to age, resident doctors aged (24–26) were significantly more exposed to verbal violence (*p* = 0.019). Men resident doctors were more frequently physically assaulted in comparison with women, which was statistically significant (*p* < 0.0001).

**Table 2:** Association between exposure to WPV and demographic variables

	Verbal violence		Physical violence	
	*n* (%)	*p* value	*n* (%)	*p* value
Age				
24–26	396 (88.2)	0.019	83 (18.94)	0.917
27–29	462 (83.09)		108 (19.42)	
30–35	97 (79.51)		24 (19.67)	
Sex				
Female	369 (38.48)	0.347	48 (10.86)	< 0.0001
Male	586 (85.55)		167 (24.38)	
Specialty				
Anesthesiology	11 (50)	< 0.0001	3 (13.64)	< 0.0001
Dermatology	67 (82.72)		7 (8.64)	
ENT	47 (79.66)		11 (18.64)	
Emergency medicine	24 (96)		9 (36)	
Family medicine	0 (0)		0 (0)	
Internal medicine	317 (86.85)		90 (24.66)	
Obstetrics	94 (93.07)		18 (17.82)	
Oncology	7 (43.75)		1 (6.25)	
Ophthalmology	40 (63.49)		2 (3.17)	
Pathology	2 (18.18)		1 (9.09)	
Pediatrics	117 (97.5)		20 (16.67)	
Psychiatry	2(100)		0 (0)	
Radiology	15 (75)		0 (0)	
Surgery	212 (89.08)		53(22.27)	
Year of residency				
Second	314(87.22)	0.049	76(21.11)	0.321
Third	296 (83.85)		68 (19.26)	
Fourth	193 (80.75)		35 (14.64)	
Fifth	122 (90.37)		73 (20)	
Sixth	24 (75)		6 (18.75)	
Seventh	6 (75)		3 (37.5)	
Related authority				
Ministry of Higher Education	434 (83.3)	0.457	77 (14.72)	< 0.001
Ministry of Defense	119 (85.61)		25 (17.99)	
Ministry of Health	402 (86.08)		113 (24.3)	
Residency province				
Al-Sweda	20 (86.96)	0.009	2 (8.7)	< 0.001
Aleppo	164 (87.23)		53 (28.19)	
Damascus	405 (88.04)		71 (15.43)	
Hama	28 (77.78)		8 (22.22)	
Al-Hasakah	26 (78.79)		12 (36.36)	
Homs	89 (82.41)		22 (20.37)	
Latakia	194 (82.2)		37(15.68)	
Tartous	29 (67.44)		10 (23.26)	
Marital status				
Married	313 (80.26)	0.002	72 (18.46)	0.702
Not married	642 (87.11)		143 (19.4)	

The frequency of violent episodes was statistically significantly different among the various specialties (*p* < 0.0001): psychiatry, pediatrics, emergency medicine, and obstetrics were the specialties with the highest frequency of verbal violence, while physical violence was more frequently observed in resident doctors whose specialty was emergency medicine (*p* < 0.0001). With regard to physical violence, resident doctors working in MoH hospitals had the highest rates (*p* < 0.001). For the geographical distribution, Damascus had the highest level of verbal violence (*p* = 0.009) and Al-Hasakah had the highest level of physical violence (*p* < 0.001).

### Verbal and physical violence characteristics

Table 3 displays the characteristics of workplace violence.

**Table 3:** Verbal and physical violence characteristics

Variables	Verbal violence, *n* (%)	Physical violence, *n* (%)
Violence frequency	955 (84.74)	215 (19.08)
Aggressor sex (multiple answers)		
Female	659 (69)	67 (31.16)
Male	861 (90.16)	189 (87.91)
Aggressor (multiple answers)		
Associates (family/friends)	856 (89.63)	178 (82.79)
Patient	340 (35.6)	50 (23.26)
Colleagues	461 (48.27)	19 (8.84)
Nurses	378 (39.62)	26 (12.09)
Others	61 (6.39)	1 (0.47)
Accident time (multiple answers)		
Morning (8:00–16:00)	779 (81.15)	89 (40.27)
Evening (16:00–24:00)	653 (68.02)	109 (49.32)
Night (24:00–8:00)	501 (52.19)	112 (50.68)
Accident location (multiple answers)		
Emergency department	790 (82.72)	183 (85.12)
Clinic	414 (43.35)	31 (14.42)
Corridor	250 (26.18)	50 (23.26)
Patient room	360 (37.7)	38 (17.67)
Others	44 (4.6)	0 (0)
Violence causes (multiple answers)		
Delay in medical care/long waiting time	765 (80.02)	134 (62.33)
Refusing patient's admission to the hospital	480 (50.21)	88 (40.93)
Alcohol or drug addict patient	170 (17.78)	26 (12.09)
Lack of information about resident doctors' responsibilities and authorities	653 (68.31)	121 (56.28)
Psychological problems	147 (15.38)	26 (12.09)
Others	44 (4.60)	6 (2.79)

Generally, violence whether it is verbal or physical was mainly perpetrated by men and patients’ associates. 81.15% of the verbal violent accidents happened in the morning, while 50.68% of the physical violent accidents happened at night. The study has shown that the emergency department was the most common location for both verbal and physical violence (*n* = 790; 82.72%, *n* = 183; 85.12%, respectively). The respondents indicated that delay in waiting/care time has been causally implicated in both verbal (80.02%) and physical (62.33%) violent incidents.

### WPV prevention strategies

**Fig. 1:**
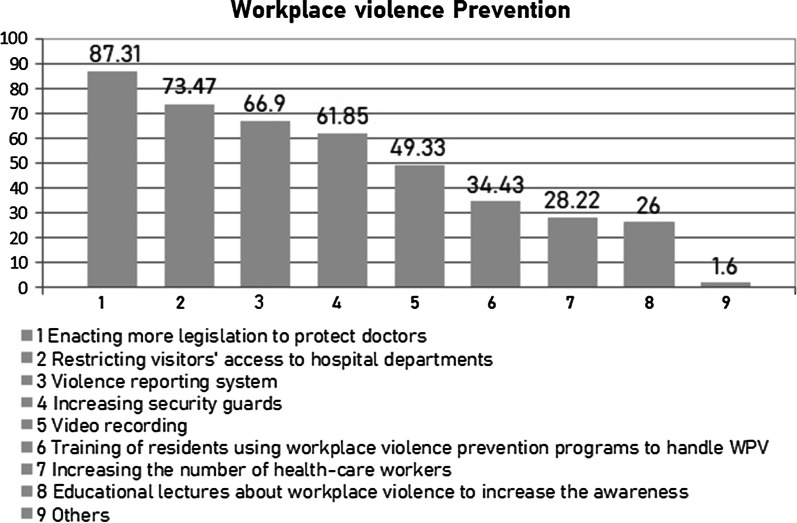
Resident doctors’ perceptions of strategies to prevent workplace violence (multiple answers)

Figure 1 shows the resident doctors’ perceptions of strategies to prevent workplace violence, which reflect a lack of violence prevention and management measures in the studied hospitals. Most resident doctors (87.31%) in this survey suggested enacting more legislation to protect doctors as the best solution to reduce WPV. Restricting visitors' access to hospital departments (73.47%), violence reporting system (66.90%), increasing security guards (61.85%), video recording (49.33%), training of resident doctors using workplace violence prevention programs to handle WPV (34.43%), increasing the number of healthcare workers (28.22%), and educational lectures about workplace violence to increase the awareness of this issue (26.00%) were the other solutions named.

### Association between WPV and psychological outcomes

Table 4 shows the correlations among each type of violence and resident doctors’ health outcomes. Verbal and physical violence were significantly positively correlated with each of the depression and stress scales’ items, and significantly negatively correlated with subjective sleep quality and subjective health.

**Table 4:** Spearman’s correlations among each type of workplace violence and health variables

	Verbal violence		Physical violence	
	Spearman’s rho	p value	Spearman’s rho	p value
General health	− 0.3891	< 0.0001	− 0.2881	< 0.0001
Sleep	− 0.3314	< 0.0001	− 0.0824	0.0111
Stress handling	0.3775	< 0.0001	0.2397	< 0.0001
Overall stress	0.3931	< 0.0001	0.3028	< 0.0001
Sadness	0.2995	< 0.0001	0.2021	< 0.0001
Loss of confidence	0.2050	< 0.0001	0.1700	< 0.0001
Guilt	0.2030	< 0.0001	0.1164	0.0001
Loss of interest	0.3665	< 0.0001	0.2614	< 0.0001

## Discussion

### Prevalence and characteristics of WPV against resident doctors in Syria

To the best of our knowledge, this is the first study in Syria to shed light on WPV, its related factors, and its psychological impact.

Based on the findings of the present study, 84.74% of Syrian resident doctors had encountered at least one type of violence during their shifts in the last 12 months prior to the survey. This is much more than that reported by previous studies [14, 24–27].

The diversity of assessment scales and different geographical location may explain the discrepancy between these studies. This could also be linked to the ongoing state of war that has in various ways eroded healthcare services. 57% of public hospitals are not functioning or are only partially functioning, placing a mounting burden and crowding on the surviving functioning hospitals.[28]

Training, planning, and monitoring have been impacted by declining numbers of senior professors, leaving resident doctors with destitute preparation or guidance and under conditions that force them to work beyond their knowledge and expertise to fill the shortage of services gap [29], and have less time to build rapport with patients, deteriorating the doctor–patient relationship.

In addition, incoherence of leadership and loss of validity of healthcare systems, due to conflict and uncertainty, are seriously undermining health policy-making frameworks themselves [30].

Furthermore, in our study, all resident doctors were working in public institutions where long waiting time is prevailing and viewed as the key factor for violence (80.02% and 62.33%, respectively, for VA and PA). One study reported that working in a public institution was significantly associated with exposure to verbal and physical abuse [31].

The findings demonstrated that resident doctors were more likely to experience verbal violence (84.74%) followed by physical violence (19.08%). This finding is consistent with studies done elsewhere [19, 32, 33]. Because in intense situations, patients usually first display rage as verbal violence then switch to intimidation and eventually show physical violence.

Frequencies of physical and verbal violence were higher in male resident doctors, and this difference was statistically significant in the case of physical violence. This finding agrees with other studies [8, 26, 34] and with literature review [35]. This may be attributable to “*the cultural norms of avoiding ‘observed’ violence against females*” [8].

A significant association was found between the resident's doctor's young age and exposure to verbal violence. This finding is in line with a previous report [36], and could be attributed to the fact that senior resident doctors have better communication skills and are more professional in dealing with nervous and agitated clients. Also, this patient-initiated violence might be due to the lack of medical management skills by junior resident doctors and the respect of patients for an older doctor. Senior resident doctors were slightly more exposed to physical violence, but there was no significant association.

In coherence with other studies [19, 20, 37], the results showed that patients’ associates were the most common source of verbal (89.63%) and physical (82.79%) violence. This seems to be related to that in Syria, as in most Asian countries, the relatives unnecessarily stay in the patient’s ward close to healthcare workers interfering with doctors’ decisions and tasks. They might also request an urge of care delivery or more medical attention.

It is noteworthy that resident doctors encountered verbal violence by their colleagues (48.27%) more than by patients (35.6%), which was found to be higher than previous study [27]. This could be attributed to the ‘pyramidal system’ of residency programs in Syria in which senior resident doctors—with the relative absence of supervisors in public hospitals—could determine juniors’ roles and comment on the lack of competency of them, which juniors may perceive as verbal violence. Besides, understaffing and the impact of stress in this very difficult working condition may be other possible factors [10].

The level of physical and verbal violence was higher among resident doctors specialized in hospitals affiliated to the Ministry of Health and this difference was statistically significant in the case of physical violence. MoH hospitals are disseminated all around Syria, opposed to MoHE hospitals that are only located in four provinces. This would generate a disparity in the allocation of resources. According to WHO report 2018, the number of resident doctors in MoH hospitals were 3,639 compared to 2,173 at MoHE hospitals [38]. Hence, MoH hospitals could suffer from a low doctor–patient ratio and insufficient institutional infrastructure compared to MoHE hospitals, which could increase patient dissatisfaction and set a lower threshold for WPV.

Additionally, there was a significant relationship between verbal violence and being a resident doctor in Damascus province. Damascus is the capital city of Syria and has the largest number of well-equipped hospitals which patients from other cities seek (21% of the overall Syrian health workload during 2018 was in Damascus) [38] to get better medical management, leading to more crowding and increasing waiting time in hospital departments, therefore, conflicts arise and WPV occurs.

### The negative effects of WPV on resident doctors’ psychological status

The medical residency is recognized as one of the most stressful and exhausting periods in a doctor’s life [39]. Syrian resident doctors have a high baseline of chronic stress as demonstrated in a recent study [40]. Moreover, due to the large number of doctors emigrating abroad [29], the remaining ones suffer from an increased workload and long working hours to compensate for the shortage of human resources. Prolonged working hours is a known risk factor for stress [41]. In addition, low financial income is another risk factor [42]. In our study, only 16.95% of participants considered salary as the only income source and many of them reported having an extra job or required financial support from their families, which might make them lose their independence. That being said, workplace violence does nothing but add oil to the fire and pose an additional threat to mental well-being [43], job performance [44], job satisfaction [24], and self-esteem of doctors, and they begin to question the worth of their work and profession while providing medical service.

According to the correlation analysis, both verbal and physical violence threatened the psychological well-being of resident doctors, which is consistent with other findings [45–47]. Interestingly, verbal violence correlated stronger than physical violence with all health outcomes variables, possibly because verbal violence occurs through a frequent basis and its intensity accumulates to become comparable with or even greater than physical violence, which occurs less frequently.

WPV also affects job motivation and contributes to less empathy and a decline in enthusiasm, which further complicates the poor relationship between Syrian doctors and patients and increases the doctors’ possibility of leaving the job. Research showed that WPV is a significant predictor of turnover intention [48] and depressive symptoms [49] in Chinese doctors. More than that, depressed resident doctors made significantly more medical errors than their non-depressed peers [49] which put patient’s safety at risk. If violence persists, more resident doctors will be pressured to abandon their jobs and the safety of the public will continue to deteriorate.

### Suggested strategies for tackling WPV

Efforts to limit WPV should be collaboratively built on different levels. First, at the healthcare system level, policymakers should enact more and reinforce existing legislation to protect doctors from aggressors’ behaviors, which has been the most proposed solution by resident doctors in our study (87.31%). Increasing healthcare workers’ numbers could also reduce the workload and lessen WPV. Second, at the hospital administrative level, respondents in this study articulated a desire of limiting visitors' access, the existence of a management system for reporting and controlling violence, and better security coverage such as video recording and increasing security guards inside the hospital. These solutions may reflect the personal satisfaction of resident doctors by the presence of additional security measurements inside the workplace environment.

Resident doctors depend largely on their own knowledge and skills to keep them safe. Therefore, we recommend that managers, supervisors, and coworkers should incorporate violence management educational programs as part of the Syrian residency training program. One study showed that such programs have measurable outcomes for a less violent workplace environment and better awareness of how to deal with aggressors [50]. In addition, training in conflict management, communication skills, and de-escalating during an aggressive event is strongly recommended and need to be integrated in a structured violence prevention program, as reported in a systematic review [51].

Third, regarding colleague-initiated violence, we strongly recommend senior resident doctors to properly use the pyramidal system to maintain its prime objective in producing well-trained junior resident doctors rather than abusing it and taking an opportunity to practice WPV against juniors. That could be strengthened if supervisors spent more time tracking the tutorial process in hospitals. Finally, since the deteriorating doctor–patient relationship is one of the key triggers of aggression towards doctors, they should devote more attention to improve their communication skills to create a more harmonious work environment [52].

All efforts against workplace violence should be developed with the prime goal of improving patient care without compromising staff safety.

### Strengths and limitations

The major strengths of this study include its large sample size in relation to the number of Syrian resident doctors. The annual Health Resources and Services Availability Monitoring System (HeRAMS) report for 2018 showed that the total number of resident doctors in MoH and MoHE hospitals from the 8 approached provinces was 5468 [38]. Thus, we assume that our sample approximately represented 18.07% (988/5468) of the resident doctors working in MoH and MoHE hospitals. However, we could not afford reliable data regarding the number of resident doctors in MoD hospitals. Also, this is the first study in Syria to investigate workplace violence and spot a light on this issue, which may help explain the high response rate and show the interest of resident doctors in this issue. Our findings will help strengthen the Syrian health profile and offer a good guide for hospital management and policy-making, so that regulations can be adopted in this regard. Furthermore, this study investigated the association between workplace violence, and resident doctors’ health status in terms of stress, depressive symptoms, general health, and sleep quality, which has important implementations in medical training and research. However, this study also has various limitations. First, the survey used to assess WPV in this study is not a validated tool and was based on literature review. The methods of measurement of sleep quality and general health were also poor. Second, according to this study design, which was an adequate and efficient way to assess the prevalence, but restrains our ability to establish a true cause and effect relationship between variables. Third, this survey is based on self-reported data, which may have led to recall and report bias. Fourth, sexual violence was not addressed in this analysis due to cultural reasons and priorities. Hence, it avoids unrealistic data and focuses on other types of violence. Fifth, we were not able to reach all of the Syrian provinces at the time of the study due to war circumstances. Finally, despite the large sample size of participants from multiple provinces, any convenient sample is vulnerable to sampling bias that may impact the generalizability of research outcomes.

## Conclusion

To the best of our knowledge, this is the first study to examine WPV among resident doctors in war-torn Syria. Syrian doctors work in an environment affected directly and indirectly by the war. The shortage of medical equipment and drugs due to restrictions and low socioeconomic status, and the emigration of well-qualified physicians made it difficult for remaining doctors to respond to the growing public needs that compromised the doctor–patient relationship and the standard of treatment given, making doctors more vulnerable to WPV and its adverse effects. The findings indicate 84.7% of participants reported that they had experienced at least one type of verbal or physical violence during their shifts in the last 12 months prior to the survey. This study also showed that WPV was positively associated with psychological stress and depressive symptoms, and negatively associated with sleep quality and overall general health of Syrian resident doctors. Therefore, in order to prevent WPV and ensure resident doctors’ psychophysical stability, it is necessary to implement legislation, develop a violence reporting system, and provide violence prevention training for resident doctors.

## Data Availability

The data analyzed during the current study are available from the corresponding author on reasonable request.

## Abbreviations

WPV: Workplace violence
MoHE: Ministry of Higher Education
MoH: Ministry of Health
MoD: Ministry of Defense
